# AR3D: Attention Residual 3D Network for Human Action Recognition

**DOI:** 10.3390/s21051656

**Published:** 2021-02-28

**Authors:** Min Dong, Zhenglin Fang, Yongfa Li, Sheng Bi, Jiangcheng Chen

**Affiliations:** 1 School of Computer Science and Engineering, South China University of Technology, Guangzhou 510006, China; hollymin@scut.edu.cn (M.D.); albumye@gmail.com (Z.F.); liyongfa1994@sina.com (Y.L.); 2 Key Laboratory of Big Data and Intelligent Robot, Ministry of Education, Guangzhou 510006, China; 3 Shenzhen Academy of Robotics, Shenzhen 518057, China; jiangcheng.0502@163.com

**Keywords:** action recognition, convolutional neural network, attention mechanism, residual, 3D

## Abstract

At present, in the field of video-based human action recognition, deep neural networks are mainly divided into two branches: the 2D convolutional neural network (CNN) and 3D CNN. However, 2D CNN’s temporal and spatial feature extraction processes are independent of each other, which means that it is easy to ignore the internal connection, affecting the performance of recognition. Although 3D CNN can extract the temporal and spatial features of the video sequence at the same time, the parameters of the 3D model increase exponentially, resulting in the model being difficult to train and transfer. To solve this problem, this article is based on 3D CNN combined with a residual structure and attention mechanism to improve the existing 3D CNN model, and we propose two types of human action recognition models (the Residual 3D Network (R3D) and Attention Residual 3D Network (AR3D)). Firstly, in this article, we propose a shallow feature extraction module and improve the ordinary 3D residual structure, which reduces the parameters and strengthens the extraction of temporal features. Secondly, we explore the application of the attention mechanism in human action recognition and design a 3D spatio-temporal attention mechanism module to strengthen the extraction of global features of human action. Finally, in order to make full use of the residual structure and attention mechanism, an Attention Residual 3D Network (AR3D) is proposed, and its two fusion strategies and corresponding model structure (AR3D_V1, AR3D_V2) are introduced in detail. Experiments show that the fused structure shows different degrees of performance improvement compared to a single structure.

## 1. Introduction

With the continuous development of deep neural networks and the promotion of human action recognition application scenarios, the research into human action recognition based on deep neural networks has become a key field in recent years. At present, deep neural networks are mainly divided into two branches in the field of video-based human action recognition: one is the use of 2D CNN for feature extraction, represented by Two-Stream CNN [1], which uses two 2D CNN models to extract and classify the spatio-temporal features of RGB pictures and optical flow pictures and then uses the SVM classifier to fuse and identiy the results; the other approach directly extracts the spatio-temporal features of the video sequence through 3D CNN [2], such as the classic C3D [3] model, and then uses the Softmax classifier for classification.

However, the existing work still has some shortcomings, which are mainly reflected in the following aspects: (1) The extraction of spatio-temporal features with the Two-Stream CNN model is independent, and it is easy to ignore its intrinsic connection, which affects the final performance of recognition; (2) When using the Two-Stream network to recognize human action, it is generally impossible to use a single RGB image. It is necessary to preprocess the RGB picture to extract time series features, such as the extraction of optical flow and the representation of motion trajectories, which increases the cost of model training indirectly. (3) Although the 3D CNN can extract spatio-temporal features of video sequences, compared with a 2D convolution operation, the model parameters of the 3D convolution operation increase exponentially, which makes it difficult to train and transfer the model; (4) The 3D convolution operation cannot distinguish between background features and human action features during feature extraction, and at the same time, the associated features before and after the series data are not captured, which makes the recognition of the model vulnerable to environmental factors. This reduces the recognition performance of the model.

With the aim of resolving the the above shortcomings, in this article, we use 3D CNN as a basis combined with residual structure and attention mechanism to improve upon the existing 3D CNN model and propose our human action recognition models. To summarize, our contributions in this paper are as follows: (1) We design a model for shallow and deep parts, extract the shallow features and deep features separately; (2) We improve the 3D residual module by decoupling the 3D convolution kernel; (3) The 3D attention mechanism is used to reduce the sensitivity of the model to changes in the background environment; (4) The experiment explores different fusion methods of the 3D residual module and the 3D attention mechanism module; (5) With only RGB images as input and without large-scale pre-training, the accuracy of the our models reaches a relatively high level.

## 2. Related Works

### 2.1. Human Action Recognition

With the continuous development of industries such as intelligent surveillance, human–computer interaction and video retrieval, the research into video-based human action recognition has attracted the considerable attention of the academic community. In recent years, the CNN has achieved success in various fields; not surprisingly, human action recognition methods based on CNNs also have proven to be very effective and are becoming a key area of research.

Simonyan et al. [1] first proposed and used Two-Stream CNN to classify single-frame RGB pictures and multi-frame optical flow pictures, respectively, and then merged two scores to obtain the final classification result. Their experimental results show that CNN trained on dense frames of optical flow images can bring significant performance improvements for action recognition tasks [1,4,5,6]. Wang et al. [4,7] proposed a Temporal Segment Network (TSN) and combined the sparse sampling strategy to divide and sample the video in the time domain to make up for the shortcomings of Two-Stream CNN in long-term time series modeling; Feichtenhofer et al. [5] proposed a method of spatio-temporal feature fusion, which solved the problem that the extractions of spatial and temporal features in Two-Stream CNN are independent.

In addition to Two-Stream CNN, 3D CNN is another important method for human action recognition. Ji et al. [2] prsented a method that captures the spatio-temporal features of multiple adjacent picture frames by performing 3D convolution operations, triggering the application of 3D CNN in human action recognition. Subsequently, Tran et al. [3] proposed the C3D model for video feature extraction and verified its effectiveness. In order to further improve performance, Tran et al. introduced the ResNet structure on the basis of the C3D model and proposed the Res3D model [8], but it led to the problem of increasing the amount of parameters; Ullah et al. [9] combined CNN and deep bidirectional LSTM (DB-LSTM) networks and proposed a new human action recognition method, which is able to learn features from long-term sequences and exhibits competitive performance compared with other state-of-the-art action recognition methods.

### 2.2. Attention Mechanism

The attention mechanism in deep learning refers to the mode of human attention thinking, which is able to quickly retrieve high-value information from massive amounts of information using limited attention resources. In recent years, the attention mechanism has been used in various network models and research fields [10,11,12,13,14,15]. Jaderberg et al. [16] proposed the spatial attention mechanism, which transforms the spatial information in the original image into another space and retains its key information. Their experimental results show that the method can improve model performance effectively. Hu et al. [11] proposed a kind of channel attention model (SENet) that assigns attention weights to the channels of the input feature map, so that the model can learn the importance of different channels’ features. Their experiments show that the SE module can achieve a significant performance improvement with a small additional computing cost. Inspired by the above two works [11,16], Woo et al. [12] designed CBAM, a spatial and channel hybrid attention module, which can be seamlessly integrated into any CNN architecture and perform end-to-end training with the basic CNNs.

However, the spatial attention mechanism, SENet and CBAM all mainly highlight the features of action from the current frame itself, without considering the internal relationship between consecutive frames. Therefore, this article addresses this shortcoming and proposes a 3D attention mechanism (see Section 3.3) to capture this relationship and strengthen the representation of the global features of human action.

### 2.3. Residual Learning

As the depth of the neural network model continues to increase, the model becomes prone to degradation and the gradient disappears. In order to solve these problems, He et al. [17] proposed a residual structure. In recent years, many articles have used residual learning for human action recognition tasks. Feichtenhofer et al. [6] proposed a residual-based Two-Stream network for human action recognition, with one stream used for learning appearance features and the other for motion features. After that, the network fuses the information learned by two separate streams, so their network can finally achieve competitive performance. Qiu et al. [18] proposed a variety of pseudo 3D structures (P3D) by decoupling 3D convolution into 2D spatial convolution and 1D temporal convolution and placing them in the ResNet block. This means that 2D spatial convolution can use the fine-tune model on the ImageNet dataset for transfer learning. As a result, the classification speed and accuracy can be greatly improved. Wang et al. [10] proposed the Residual Attention Network. Similar to our work, Wang et al., integrated attention modules into residual blocks to take advantage of both residual learning and the attention mechanism. However, their work was designed for image classification tasks and they did not consider the residual learning of temporal information in videos. In this article, we extract the features from video in both spatial and temporal dimensions and fuse the 3D attention module to the 3D residual module, improving the performance for human action recognition.

## 3. Materials and Methods

### 3.1. Data and Pre-Processing

UCF101 and HMDB51 are two commonly used benchmark video datasets and were utilized in this study. Both of them are available online [19,20]. UCF101 covers some basic actions in daily life, and its background is close to an actual scene. Therefore, UCF101 is also the most commonly used comparative test dataset in the field of human action recognition. According to the official division standard, the 101 categories of the UCF101 data set can be divided into five categories: basic human movements, human-human interaction, human-object interaction, musical instrument performance and sports. HMDB51 contains 51 types of behavior actions, with a total of 6849 videos, each of which contains at least 101 video clips (the number of videos in the training data is 70, the number in the test data is 30). The samples of this dataset mainly come from movies, and some come from video websites such as YouTube. The 51 types of actions contained in HMDB51 can be divided into five categories by official division standards: general facial movements, facial movements and object operations, general body movements, human-object interaction and human-human interaction.

The deep learning model proposed in this paper belongs to the category of 3D CNN, which generally uses continuous multi-frame RGB pictures as the input data of the model, so the original video dataset needed to be preprocessed before model training. This mainly included the following steps: (1) Video frame conversion: A video frame conversion program was used to obtain picture frames for all videos in each category and save them in the corresponding video folder, and the total number of picture frames after each video conversion were counted; (2) Dataset division: We divided the UCF101 and HMDB51 datasets according to the five categories mentioned above, meaning that the training record was regenerated for each video by using the labeling program. The format was as follows: picture frame folder path → category number; (3) Making the dataset: We used the the Dataset API provided by TensorFlow to encapsulate the data set corresponding to step (2) as a dataset object. The encapsulation process included operations such as random scramble, data enhancement, cropping and normalization of the image data. The main purpose of this was to enhance the diversity of the dataset and prevent the model training from overfitting.

### 3.2. 3D Deeper Residual Network (R3D)

**Figure 1:**
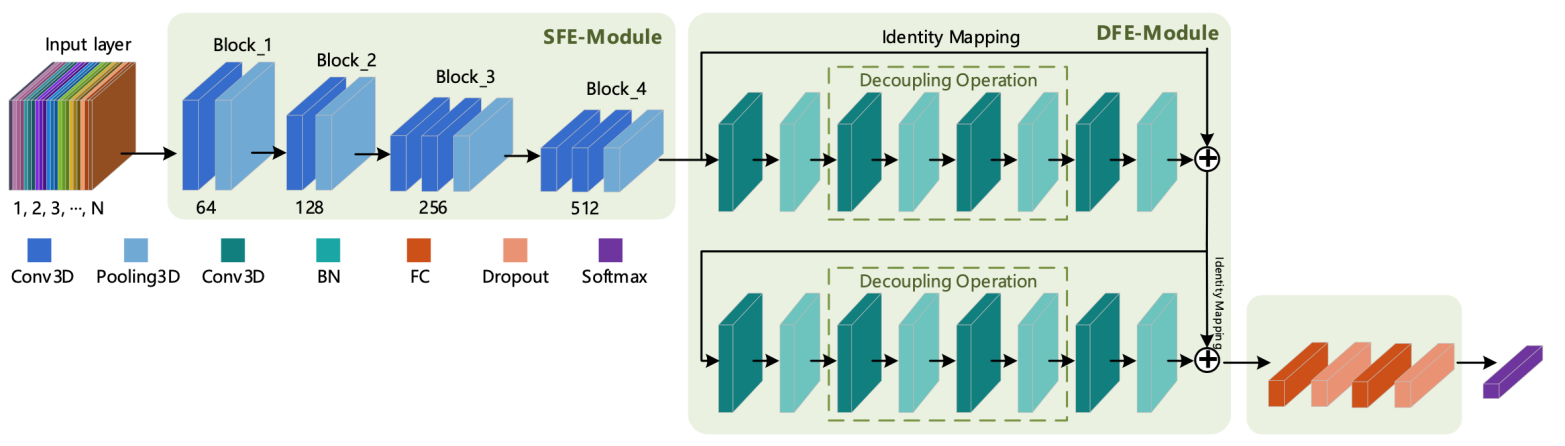
Architecture of the Residual 3D model (R3D).

Tran et al. [3] proposed the C3D model to capture spatio-temporal features, but the overall shallow structure of the C3D model makes it difficult to extract deep action features. Thus, Tran et al. proposed the Res3D [8] model based on the C3D model and residual structure, but it led to the problem of excessive model parameters. In order to benefit from the advantages of these two models and overcome their respective disadvantages, in this section, we present our proposal and design of a 3D Shallow Feature Extraction Module (3D SFE-Module) to extract the shallow feature information of a video sequence and the design of a residual module suitable for 3D convolution according to the characteristics and properties of the residual structure to extract richer deep-layer action features. The 3D Deeper Residual Model (R3D) is formed by combining the 3D SFE-Module and the 3D residual module. The overall structure of the corresponding model is shown in Figure 1.

It can be seen from Figure 1 that the R3D model consists of an input layer, a feature extraction layer, a fully connected layer and a classification layer. The input layer is composed of n∈[1,2,3,⋯,N] frames of continuous RGB (or other format) pictures, while the feature extraction layer contains two parts: a Shallow Feature Extraction Module (SFE-Module) and a Deep Feature Extraction Module (DFE-Module). The DFE-Module is the focus of research and improvement in this article (the DFE-Module uses the Residual 3D Module in this section and uses the fusion of 3D Attention and 3D Residual Modules in Section 3.4). The fully connected layer consists of the fully connected operation and the neuron inactivation operation (Dropout). The introduction of Dropout was mainly done to increase the probability of the random inactivation of the model and reduce the number of connections between neurons, thereby preventing the risk of model overfitting. The last layer is the classification layer; the human action recognition studied in this paper produced a multi-class output, so the Softmax classifier was selected in the classification layer.

Since the structures of the input layer, fully connected layer and classification layer of the deep learning-based human action recognition model were basically the same, the following text mainly focuses on the principle and corresponding structure of the feature extraction module. In this section, we first introduce the 3D SFE-Module and the 3D Residual Module in the R3D model.

#### 3.2.1. Three-Dimensional SFE-Module

**Figure 2:**
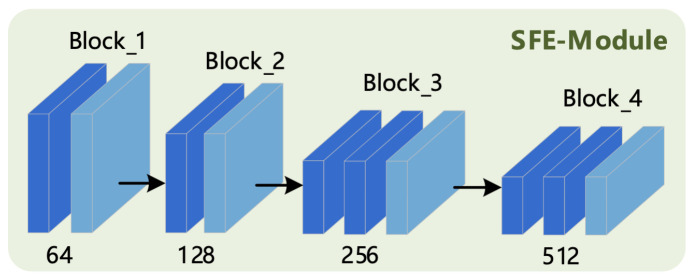
Three-Dimensional Shallow Feature Extraction (SFE) Module.

The 3D SFE-Module refers to the structural design of the C3D model [3]; it takes continuous multi-frame original picture sequences as an input and then passes these through its internal convolutional layer and pooling layer to extract the shallow feature information of human action. The final output is used as the input of the DFE-Module. The corresponding structure is shown in Figure 2.

It can be seen from Figure 2 that the module consists of four small blocks, which each block containing one or more 3D convolution operations and one 3D pooling operation; all convolution and pooling operations use the “SAME” filling mode, and the specific parameter configuration is shown in Table 1.

**Figure 3:**
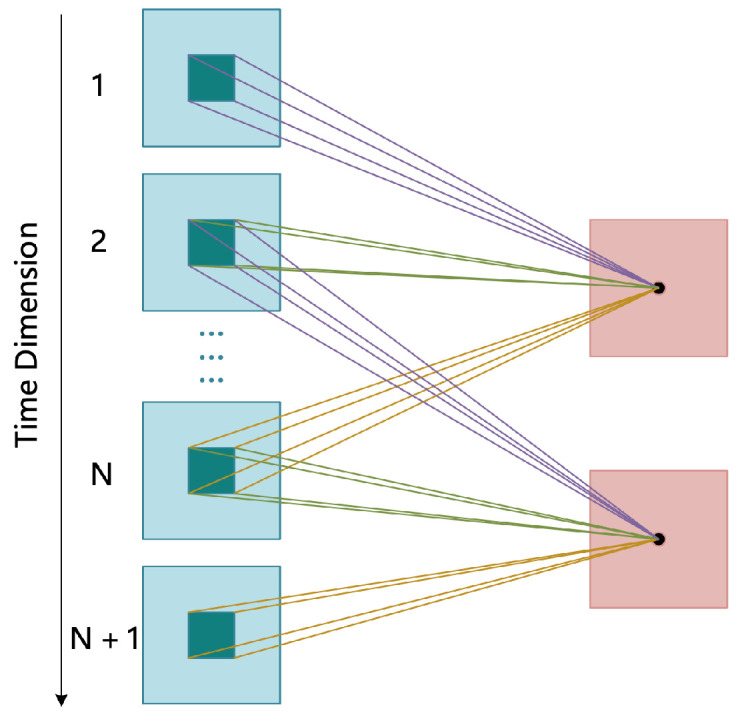
Three-dimensional convolution temporal feature extraction.

In the designed 3D SFE-Module, two operations were mainly carried out—3D convolution and 3D pooling—to extract shallow spatio-temporal features. The 3D convolution operation refers to the use of a three-dimensional convolution kernel (the size of the convolution kernel used in this article was 3×3×3) to extract the features of the input continuous multi-frame picture sequence. This is mainly because the input picture sequence contains not only the features of the spatial dimension but also contains the temporal information of the time sequence. In contrast to the 2D convolution operation, 3D convolution adds the feature extraction operation in the temporal dimension. The temporal feature extraction diagram is shown in Figure 3.

#### 3.2.2. Three-Dimensional Residual Module

The 3D residual module takes the shallow features extracted by the 3D SFE-Module as input and then passes them through the convolutional layer and the normalization layer to extract the richer deep feature information.

**Figure 4:**
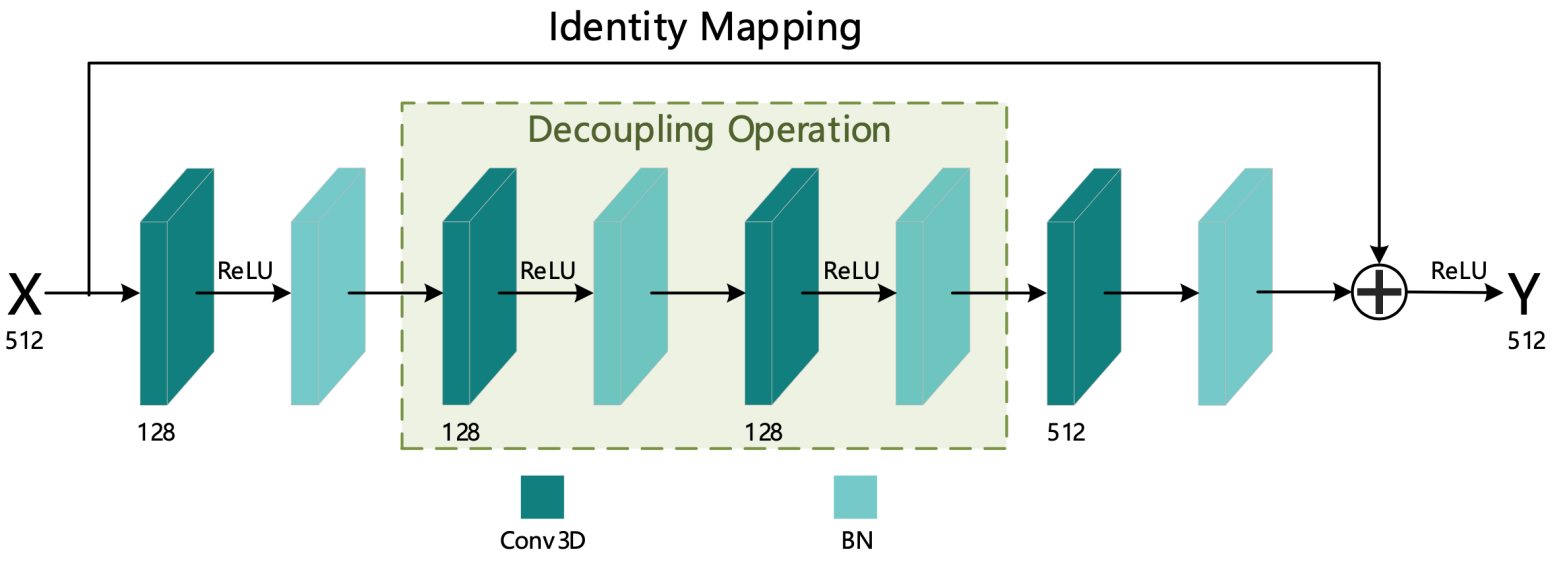
Three-dimensional residual structure.

To increase the number of network layers and prevent the gradient of the deep network from disappearing, this article refers to the ordinary residual structure [17]; to this end, we designed a 3D residual structure suitable for spatio-temporal feature extraction. The specific structure diagram is shown in Figure 4, and the Residual 3D Module is composed of two such residual structures (as shown in Figure 1).

As can be seen from Figure 4, the 3D residual structure is composed of four convolutional layers, four normalization layers and an identity transformation addition operation. All convolution operations used the “SAME” filling mode, and the specific parameter configuration of the convolution layer is shown in Table 2.

**Figure 5:**
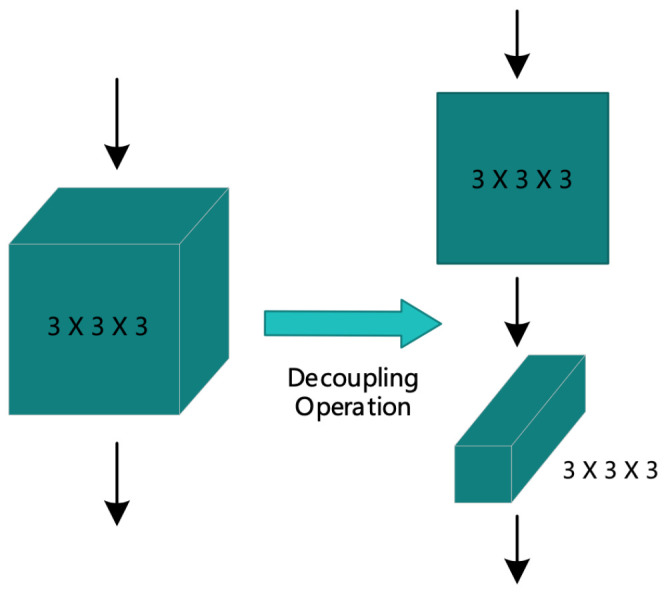
Decoupling 3D convolution kernel.

From Table 2, we can see that the design of Conv_2 and Conv_3 convolution kernel used a decoupling operation on the 3D convolution kernel 3×3×3 and decomposed it into two operations of two-dimensional spatial convolution and one-dimensional temporal convolution. This operation is more conducive to extracting features in the temporal dimension. The specific decoupling diagram is shown in Figure 5.

The normalization layer was used to normalize the input data to make the data uniform, thereby reducing the impact of data distribution on model training. At the same time, this operation can also prevent overfitting during model training. The specific operation process can be divided into the following four steps.

(1) Calculate the mean value of each input batch of data. Assuming that the batch input data are $x\in \left\{x_{1},x_{2},\cdots ,x_{n}\right\}$ and the obtained mean value is μ, the mean value can be calculated by
  (1) $$\mu =\frac{1}{n}\sum_{i=1}^{n}x_{i}$$
(2) Solve the variance $\sigma^{2}$ of each input batch of data:
  (2) $$\sigma^{2}=\frac{1}{n}\sum_{i=1}^{n}{\left(x_{i}-\mu \right)}^{2}$$
(3) Use the mean μ and the variance $\sigma^{2}$ obtained in step (1) and (2) to normalize the data to obtain its corresponding 0–1 distribution:
  (3) $$\hat{x_{i}}=\frac{x_{i}-\mu }{\sqrt{\sigma^{2}+\varepsilon }}$$
  where $\hat{x_{i}}\in \left\{\hat{x_{1}},\hat{x_{2}},\hat{x_{n}}\right\}$ is a certain datum after normalization, and ε is a small value (generally taken as $10^{-5}$), mainly used to prevent the divisor from being 0.
(4) Perform scale transformation and translation operations on the normalized sample $\hat{x_{i}}\in \left\{\hat{x_{1}},\hat{x_{2}},\hat{x_{n}}\right\}$ obtained in step (3), and finally obtain the output of the normalized layer:
  (4) $$y_{i}=\gamma \hat{x_{i}}+\beta$$
  where both the scale transformation parameter γ and the translation parameter β are learned by the neural network during training.

The identity transformation addition operation is used to add the input received by the residual structure to the output result of the residual structure after being transformed by the identity mapping, and finally the added result is applied to the ReLU activation function to increase its output nonlinearity.

### 3.3. Three-Dimensional Attention Mechanism

**Figure 6:**
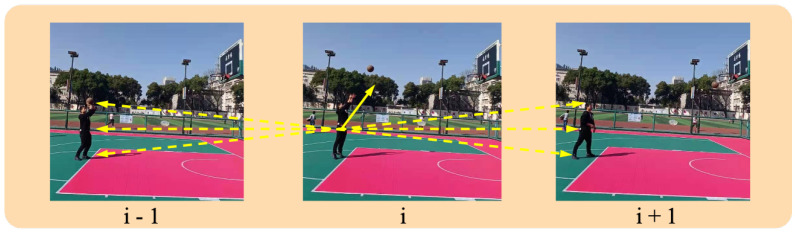
Connections between consecutive multi-frames. From the figure, we find that the current action *i* is not only affected by its own state, but may also be affected by its predecessor action i−1 and subsequent action i+1. Therefore, if the connection between the frames of the image sequence can be captured, it is possible to improve the accuracy of human action recognition.

In video-based human action recognition, since the input is a consecutive multi-frame image sequence, in addition to the features of the action itself, some connections between consecutive multi-frames will remain, as shown in Figure 6.

**Figure 7:**
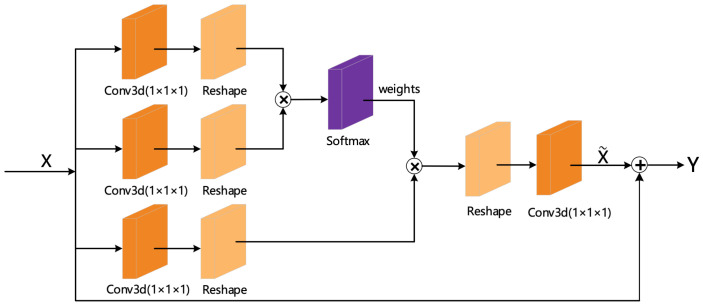
Three-dimensional attention mechanism structure.

In Section 2.2, three commonly used visual attention mechanism models are mentioned, which are mainly shown to highlight the features of action from the current frame itself, without considering the internal relationship between consecutive frames (see Figure 6). Therefore, this section addresses this shortcoming and proposes a 3D attention mechanism (for the specific structure diagram, see Figure 7) to capture this relationship and strengthen the representation of the global features of human action.

From Figure 7, we can see that the structure consists of three convolution branches and one addition operation. The convolution branch can be divided into two different paths (counting from top to bottom): the first and second convolution branches form the attention weight extraction part, while the third convolution branch is the feature extraction part.

In the attention weight extraction part, we first performed a 1×1×1 convolution operation on the input feature map to reduce parameters and calculations and then reshaped the feature map into a two-dimensional matrix through the reshape layer. Then, we performed matrix multiplication to calculate the similarity to obtain the corresponding weight value and finally used the Softmax function for normalization operation to convert the calculated result into a probability value between [0,1]. The size of the probability reflected the assigned attention size.

In the feature extraction part, we first used a 1×1×1 convolutional layer to act on the input feature map to extract the feature while reducing the size of its dimension, and then converted the feature map into a two-dimensional matrix through the reshape operation. Finally, we multiplied the result and the attention weight and output the feature map to which the attention was allocated.

In order for the 3D attention mechanism structure to be embedded in any network structure, it is necessary to ensure the consistency of its input and output dimensions. Therefore, before the final attention feature $\tilde{x}$ was output, we added a reshape layer and a 1×1×1 convolutional layer to restore the dimension of the feature map.

In the addition operation part, referring to the design of the 3D residual structure, we directly added the original input *x* received by the 3D attention structure to the attention feature map $\tilde{x}$ and then output *y* as the input of the next module of the neural network. The purpose of this design was to reduce the possibility of gradient disappearance when adding this module to the deep network.

In summary, the specific implementation of the 3D attention mechanism module can be abstracted as the following equations (see Equations (5)–(8)):

(5) $$\delta^{\prime }=Softmax\left((R\circ (Conv\circ x))⊗(R\circ (Conv\circ x))\right)$$

(6) $$\delta^{\prime \prime }=R\circ \left(Conv\circ x\right)$$

(7) $$\tilde{x}=Conv\circ \left(R\circ (\delta^{\prime }⊗\delta^{\prime \prime })\right)$$

(8) $$y=w\tilde{x}⊕x$$

where Conv ∘ means the convolution operation, R ∘ means the reshape operation, ⊗ means the matrix multiplication operation and ⊕ means the matrix addition operation.

### 3.4. Attention Residual 3D Network (AR3D)

In this section, we present the fusion of the 3D Residual Module and 3D Attention Module designed in the previous sections, creating a brand new module to capture richer deep feature information.

We proposed two fusion strategies: strategy 1 fused the 3D attention mechanism into the connection of the identity transformation of the 3D residual structure, while strategy 2 fused the 3D attention mechanism to the output of the 3D residual structure. The difference between these two fusion strategies was mainly the location at which the 3D attention mechanism was added. Thus, the models that use these two strategies are collectively referred to as the Attention Residual 3D Network (AR3D), and the model generated by strategy 1 is named AR3D_V1, while that generated by strategy 2 is named AR3D_V2.

#### 3.4.1. AR3D_V1

**Figure 8:**
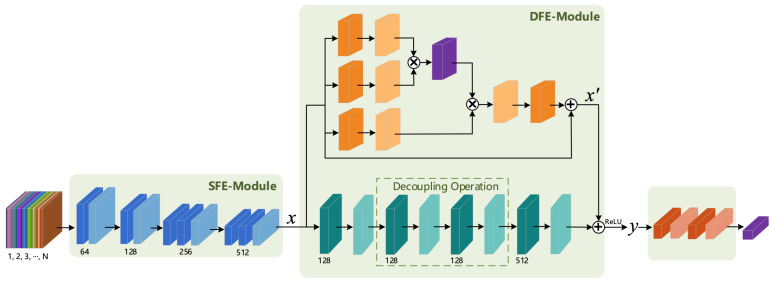
The architecture of AR3D_V1.

The DFE Module of the AR3D_V1 used fusion strategy 1 to fuse the 3D attention mechanism into the connection of the identity transformation of the 3D residual structure; the corresponding architecture is shown in Figure 8.

Assuming that the input received by this module was *x*, it passed through a 3D attention module to obtain the feature $x^{\prime }$ assigned to the attention mechanism; then, $x^{\prime }$ was added to the result of the convolutional layer output, finally obtaining the final result *y* through the ReLU function, as shown in the following equation:

(9) $$x^{\prime }=A\left(x\right)$$

(10) $$y=f_{2}\left(f_{1}\left(w_{conv}x+b_{conv}\right)+x^{\prime }\right)$$

where *A* represents the attention feature extraction operation (see Equations (5)–(8) for details), $w_{conv}\in R^{x\times y\times z}$ is the convolution kernel parameter, $b_{conv}\in R$ is the bias value and both $f_{1}$ and $f_{2}$ are ReLU activation function subscripts representing different positions.

#### 3.4.2. AR3D_V2

**Figure 9:**
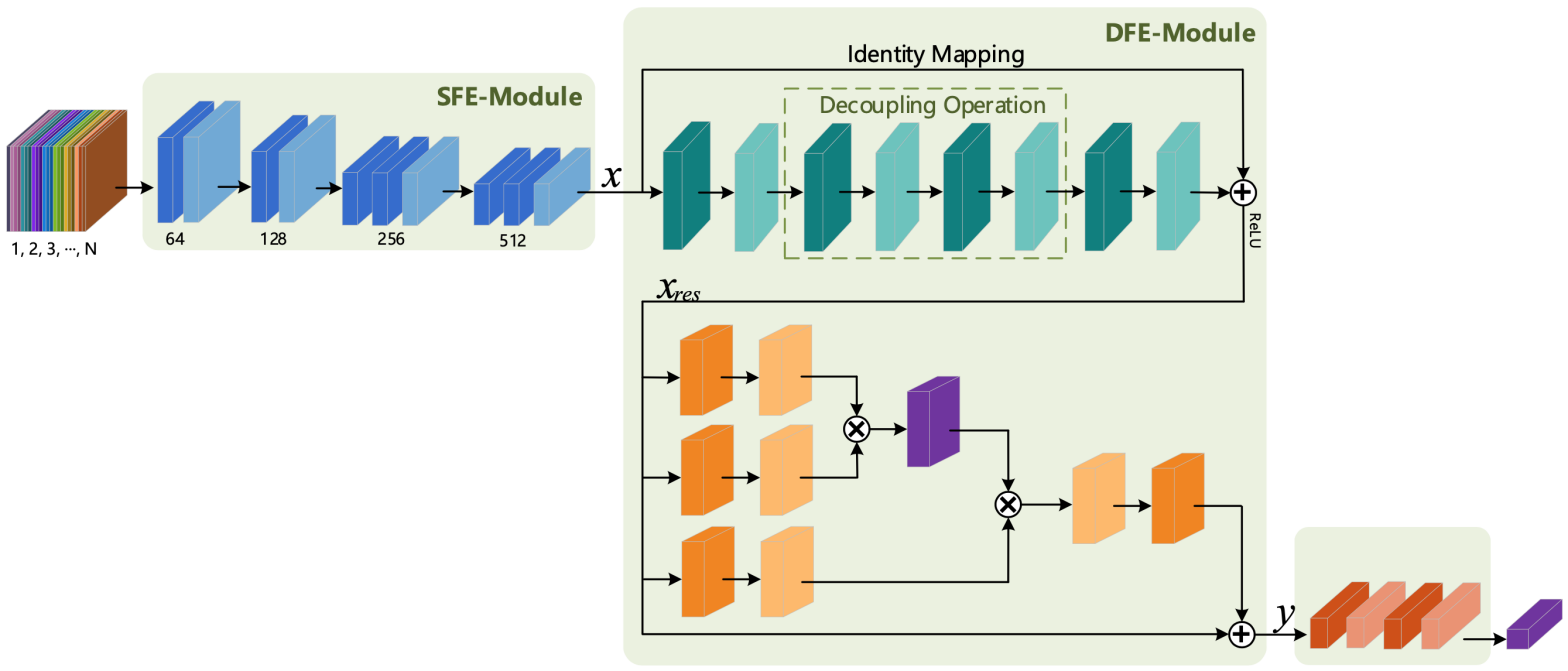
The architecture of AR3D_V2.

The DFE-Module of the AR3D_V2 model was obtained by using fusion strategy 2. The specific structure is shown in Figure 9.

From Figure 9, it is found that the deep features obtained by the 3D residual structure were then extracted by the 3D attention module. Compared with the DFE-Module in the AR3D_V1 model, the DFE-Module of AR3D_V2 did not change the identity mapping operation of the 3D residual structure, meaning the two existed in a sequential manner and allowing the extraction of the deep action features.

Assuming that the input received by this module was *x*, and then the 3D residual module output its corresponding residual feature $x_{res}$ (see Equation (11)) and then went through the 3D attention module and assigned the attention mechanism to $x_{res}$, the final output feature *y* would be obtained (see Equation (12)).

(11) $$x_{res}=f\left(w_{res}x+b_{res}\right)$$

where $w_{res}\in R^{x\times y\times z}$ represents the convolution kernel parameter in the 3D residual module, $b_{res}\in R$ is the corresponding offset value and *f* is the corresponding activation function (in this article, this is ReLU).

(12) $$y=A(x_{res})$$

where *A* represents the attention feature extraction operation, and the specific process is shown in Equations (5)–(8).

## 4. Experiment

All experiments in this article were based on the Ubuntu 18.04 bionic system, the CPU was Intel Xeon E5-2620v4 and the GPU was a GeForce RTX 2080 Ti. The development toolwasis Sublime Text3, the programming language was Python3, the image processing library was OpenCV4.1.1, and the deep learning framework was TensorFlow and Keras.

### 4.1. Experimental Training Process

The purpose of this article was to improve the performance of human action recognition models; thus, the measured indicators were based on the accuracy of recognition. Assuming that the sample size of a certain dataset is *n*, the predicted category of a certain sample $x_{i}$ is $y_{i}^{pred}$ and the true category is $y_{i}^{t}rue$; then, the calculation of its accuracy rate is shown in equation below:

(13) $$pression=\frac{1}{n}\sum_{i=1}^{n}F\left(y_{i}^{pred}==y_{i}^{true}\right)$$

where F(x) is the indicator function: if $y_{i}^{pred}==y_{i}^{true}$ is true, the return value is 1; otherwise the return value is 0.

The model in this article used the Mini-Batch Gradient Descent (MBGD) method during training, where mini-batch refers to randomly selecting a training subset from the training dataset. Assuming that a certain training set contains *n* samples and the mini-batch size is *b*, then the entire dataset is divided into $\frac{b}{n}$ mini-batches. Running a mini-batch sample is usually considered as a step towards completing the training. When all the running steps were completed, then the entire dataset was scrambled, and the above steps were repeated until the loss of the model converged or reached a satisfactory accuracy.

The input of the customized model in this article was a continuous 16-frame RGB picture; the picture size was uniformly cropped to 112×112, and the minimum batch size was set to 25. The loss function for model training was the categorical cross entropy (CE) loss function; the initial learning rate was 0.001, and the learning rate was adjusted by exponential decay, meaning that the decay factor was 0.1 and the decay step was set to 2000. The model’s optimizer was “Adam” and the early stopping threshold was set to 10.

All the parameters of the comparison model in this paper adopted the default settings in the original paper, which was done to ensure the fairness and effectiveness of the comparison experiment.

### 4.2. Experimental Comparison Model

The baseline models included 3D-ConvNet [2] and C3D [3], which are the reference standards for the performance improvement of the model proposed in this paper. Of these, 3D-ConvNet was the first to propose the concept of 3D convolution; C3D is a classic model of 3D CNN in the field of human action recognition, and the design of the SFE-Module in this article also refers to its partial structure. Therefore, it was reasonable to choose these two as the baseline models of this article.

Other models included mainly provided the principles and structures to which this article has referred in the design of the DFE-Module, as well as some successful evolution models in recent years. A summary of the comparison model is shown in Table 3.

## 5. Results

### 5.1. Performance Comparison

In Table 4, all models are divided into three areas: baseline, others and ours. The symbol “+” indicates the improved accuracy of the four models proposed in this article compared to the best of the baseline models; that is, the percentage increase compared to the C3D (three nets) model.

**Figure 10:**
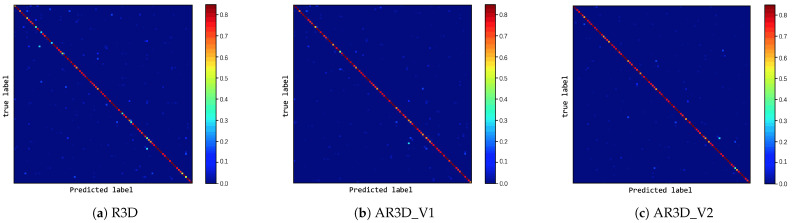
Confusion matrix of three proposed models on UCF101.

**Figure 11:**
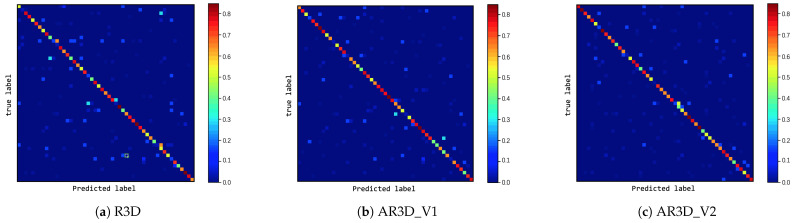
Confusion matrix of three proposed models on HMDB51.

It can be seen from Table 4 that the accuracy of three methods proposed in this article improved compared with all baseline models. With UCF101, the methods proposed in this article improved by 2.69% at least and 4.08% at best compared with the best model C3D (three nets) in the Baseline. On HMDB51, compared with C3D (three nets) in the Baseline, the methods proposed in this article improved by 4.07% at least and 6.3% at best. The confusion matrix of the three proposed methods on UCF101 and HMDB51 is given in Figure 10 and Figure 11, where the intensity of true positives (diagonal) was high for most categories, proving the efficiency of the proposed methods on the UCF101 and HMDB51 datasets. It also can be seen from Table 5 that our models had fewer parameters than C3D.

It also can be seen from Table 4 that, for UCF101, AR3D_V2 achieved the best performance among the methods using only RGB input (except for DB-LSTM, which has higher accuracy). The state-of-the-art work I3D [23] achieved very good performance on UCF101 (97.9%) and HMDB51 (80.2%) with their two-stream I3D model using both RGB and optical flow inputs; the approach is an ImageNet and Kinetics pre-trained model. When using only RGB as input and without pre-training, their results for UCF101 and HMDB51 were 84.5% and 49.8%, which were 4.78% and 2.71% worse than the AR3D_V2 model proposed in this article. Therefore, when evaluating the models, we should notice whether the models have undergone large-scale pre-training, which could play a key role in improving the accuracy of the model.

### 5.2. Efficiency Comparison

In this part, we evaluate the efficiency of our methods and compare them with other state-of-the-art methods. The experiment was performed on UCF101 and HMDB51 datasets. As shown in Table 5, the efficiencies of our methods were maintained in real-time (>25 fps). This shows that our proposed methods could obtain high-accuracy performance with lower additional computation costs. Although the I3D [23] methods which use RGB and optical flow images as input could surpass our method in accuracy, the number of parameters in their method was about four times that of ours methods, and the calculation speed of the optical flow was too slow, seriously affecting the efficiency of their models.

### 5.3. Comparison of Three Models Proposed in this Article

When the DFE Module was a 3D residual structure, the R3D model in this article was slightly worse than the AR3D model. Our analysis showed that the 3D attention mechanism effectively improves the performance. When the DFE Module was a fusion of attention and residual information, the performance of AR3D_V2 was better than AR3D_V1, which shows that the sequential fusion of the attention mechanism and the residual structure is better than adding the attention mechanism to the connection of the identity mapping, mainly because the latter changes the characteristics of the residual and loses the feature information extracted by the Residual 3D module.

## 6. Conclusions

In this article, we propose AR3D, a 3D CNN architecture for video-based human action recognition. In this network, our approach includes a 3D residual structure and 3D attention mechanism structure. This network architecture has two advantages: one is the introduction of a 3D residual structure with decoupling operations, which enables us to build a deep 3D network structure to improve the accuracy of human action recognition; the other is the improvement of the 3D attention mechanism module, taking into account the internal relationship between consecutive frames and providing an attention mechanism for the network. Experimental results show that our AR3D model can achieve good performance.

## Figures and Tables

**Table 1:** Specific parameter configuration of each layer of the 3D SFE-Module.

Block Number	Convolutional Layer Number	Convolutional Kernel Size	Convolutional Kernel Number	Pooling Layer Number	Pooling Kernel Size
Block_1	1	3×3×3	64	1	1×2×2
Block_2	1		128		2×2×2
Block_3	2		256		2×2×2
Block_4	2		512		2×2×2

**Table 2:** Specific parameter configuration of convolutional layer of the 3D residual structure.

Number	Convolutional Kernel Size	Feature Dimension	Activation Function
Conv_1	1×1×1	128	ReLU
Conv_2	1×3×3	128	ReLU
Conv_3	3×1×1	128	ReLU
Conv_4	1×1×1	512	NAN

**Table 3:** Comparison model information summary.

Model Name	Dimension	Proposal Time	Category
3D-ConvNet [2]	3D	2013	3D CNN
IDT [21]	2D	2013	2D CNN
C3D [3]	3D	2015	3D CNN
Two-Stream [1]	2D	2015	2D CNN
VideoLSTM [22]	2D	2016	LSTM + Attention
DB-LSTM [9]	2D	2017	LSTM
Res3D [8]	3D	2017	3D ResNet
P3D-A [18]	3D	2017	3D ResNet
I3D [23]	3D	2018	3D CNN
MiCT-Net [24]	2D, 3D	2018	2D CNN + 3D CNN
3D RAN(ResNet-18) [25]	3D	2019	3D ResNet + Attention

**Table 4:** The recognition accuracy of all models on UCF101 and HMDB51.

Method	Pretraining	UCF101		HMDB51	
Baseline					
3D-ConvNet [2]	–	51.6		24.3	
C3D (1 net) [3]	–	82.3		40.4	
C3D (3 nets) [3]	–	85.2		46.2	
Others					
IDT [21]	–	86.4		61.7	
Two-Stream [1]	ImageNet	88		59.4	
VideoLSTM [22]	–	79.6		43.3	
DB-LSTM [9]	ImageNet	91.21		87.64	
Res3D [8]	–	85.8		54.9	
P3D-A [18]	ImageNet	83.7		–	
MiCT-Net [24]	ImageNet	84.3		48.1	
3D RAN (ResNet-18) [25]	–	47.6		21.3	
I3D (RGB) [23]	–	84.5		49.8	
I3D (RGB) [23]	ImageNet+Kinetics	95.4		74.5	
I3D (Flow) [23]	–	90.6		61.9	
I3D (Flow) [23]	ImageNet+Kinetics	95.4		74.6	
I3D (Two-stream) [23]	–	93.4		66.4	
I3D (Two-stream) [23]	ImageNet+Kinetics	97.9		80.2	
Ours					
R3D	–	87.89	+2.69	50.27	+4.07
AR3D_V1	–	88.39	+3.19	51.53	+5.33
AR3D_V2	–	89.28	+4.08	52.51	+6.31

**Table 5:** Model parameters and speed comparison.

Method	Params ($\times 10^{6}$)	Speed (fps)
MiCT-Net [24]	53.9	394
C3D [3]	78.4	323
P3D-A [18]	63.7	140
I3D (Two-Stream) [23]	250	14
Ours		
R3D	56.5	125
AR3D_V1	65.1	78
AR3D_V2	65.1	75
